# Reflections from Key Policy Decision-makers on Integrated Care and the Value of Decision-maker Involvement in Research

**DOI:** 10.5334/ijic.4161

**Published:** 2018-05-09

**Authors:** Karen Evison, Denis A. Roy, Fredrika Scarth

**Affiliations:** 1 Ministry of Health in New Zealand, Hamilton, NZ; 2 Institut National d’Excellence en Santé et en Services Sociaux (INESSS), Montreal, CA; 3 Ontario Ministry of Health and Long Term Care, Toronto, CA

**Keywords:** Integrated Knowledge Translation, Decision-Maker, Integrated Care, International Comparisons, Health Care Policy

## Abstract

The iCOACH study involved key health care system decision-makers from Ontario, Quebec and New Zealand. This article is written by the key decision-makers involved in the iCOACH study and discusses their motivations to engage in the research project, the value of participation and key recommendations for best practices to engage decision-makers in research projects. Suggestions for knowledge translation are identified including practical tools for decision-makers and providers to use to assess readiness to implement integrated community-based primary health care. Case study briefs with key enablers and ‘talking-points’ and infographics are similarly recommended as approaches to transfer knowledge gained from this research study.

## Introduction

Integrating Care for Older Adults with Complex Health needs (iCOACH) is a multi-year, international collaborative research program examining and comparing community-based primary health care (CBPHC) in three jurisdictions: New Zealand, Quebec, and Ontario. The objective of the iCOACH program is to develop a systematic guide to the design, implementation, and scaling-up of innovative integrated models of CBPHC.

We were invited to participate in the iCOACH project as key decision-makers in New Zealand, Quebec, and Ontario. In this article we reflect on our experience with iCOACH and provide advice on the role of decision-makers in research. We also outline suggestions for tools and products arising from the research that would be of value to decision-makers.

## Motivation to Engage in the iCOACH Project

When considering engaging in the iCOACH project, the first motivating factor was who was asking; we always want to make sure that we are connecting with people from whom we are going to learn. Being aware of some of the researchers’ previous work, we knew the importance of the request.

The second factor was that the iCOACH concept was highly topically relevant in all three of our jurisdictional contexts. Quebec has been approaching integrated care with a system-wide focus on transformation, while the Ontario “Health Links” initiative specifically targets the complex health needs sub-population. At the initiation of this research project, Ontario was also moving to further enhance service integration in primary and community care through the development of local sub-regions and structural reform that would bring home and community care and primary care services into regional planning and funding networks (LHINs). We felt that the jurisdictions could learn from one another in terms of paradigm and general approach. For New Zealand, participating in iCOACH would bring an academic perspective and evidence to support the existing theory in use at the Ministry of Health and uncover barriers to integrated care and chronic conditions management in our jurisdiction.

Finally, we considered the implications of participating in iCOACH as a professional development strategy, not only for us as individuals, but for our organizations. We are generalists; in order to take care of many different concurrent themes, we need to develop particular connections to experts in priority areas. These types of partnerships are an efficient way to connect with the leaders in specific areas.

## Value of Participation

From a topical point of view, the commonalities and patterns that emerge from the research will add to what is currently known and used in our work. For example, what are the common driving forces present in terms of the population needs and how do we approach solutions to these needs? From the iCOACH discussions we have been able to infer a number of patterns and key lessons learned that are of great interest to policy.

We hope to use this project to better appreciate the value of integrated approaches for the future of our health systems. As such, we need to translate the qualitative research into something robust around which we can build good methodologies. The value of this research for us will be to bring evidence on such questions as: What should be included in an integrated care program? How should it be managed and governed? How should clinical performance be assessed? What are the key levers for change that should be envisioned at the Ministry level or supported more locally?

One of the assets of the iCOACH project is that it is nested within identifiable jurisdictions. Learning within jurisdictions allows for comparison to others in similar contexts. Consideration of relatable peer groups accelerates improvement efforts, facilitates commitment to change, and can also impact spread and scalability.

## Translating Assets to Action

Research can also directly impact iterative policy change; decision-makers are influenced by who we meet, what we hear and whether it resonates and makes sense. Coming in to the project, we expected that early on we would have ideas and guidance on how to achieve better integration and chronic conditions management that, for example, could be put through planning guidance and simulated quickly. However, while the iCOACH research has helped change our conversation around integrated care — to influence our thinking and language around patient and carer perspectives and the role of non-registered health professionals, for example — we have not yet seen anything that can enable the rapid transformation of research into action. This is a limitation in terms of value of participation thus far.

While the iCOACH project may not have fulfilled the policy-maker’s dream of rapid prototyping and translation into action, the richness of the research design should yield significant insights. One of the particular strengths of the iCOACH project is its focus on multiple perspectives – patient, carer and family, provider, organization, and policy – to identify critical factors for successful implementation. From a policy decision-maker perspective, this framework may prove to be very helpful in identifying policy barriers to integrated care that can be addressed at the health system level, whether provincial or national, as well as areas where a more enabling national or provincial policy environment is needed to support local leadership and innovation.

## Why Involve Decision-Makers in Research

Decision-makers and knowledge-users are ambassadors of context. We learn from the research process, but we can also lend an understanding of how real life works. Research projects can often take on a siloed approach — an insular, isolated researchers’ perspective without the broader context that adds value to the work. Decision-makers can add an important dimension to academic independence with valuable perspectives as a source of variation, while still maintaining the integrity of the research teams.

Decision-makers can also add value with our ability to re-contextualize the work in specific jurisdictions so the knowledge can become a lever for improvement and transformation. Decision-makers can bring in key stakeholders to examine the findings and their policy and managerial implications to further contextualize the research. Thus, decision-makers as well as key stakeholders should be embedded in the research project to enable and support operational learning.

Ultimately, the advice and contributions of decision-makers influence system dynamics; they can become a public norm against which politicians, stakeholders, and providers can be held to account.

## Recommendations for Improvement: How to Better Engage Decision-Makers in Research

A challenge in this particular project was that one of the decision-makers was brought into the project at a late stage. Decision-makers can be the most effective if approached early on in the project during the design phase. We are not particularly useful, for example, in the review of questionnaires and similar processes, but can be very helpful upstream, to refine the research questions and hypotheses, and downstream of this phase, for example to discuss how contextual factors influence or confound the observed results and to inform how research findings can be most effectively translated and communicated to broader decision-maker audiences.

A second challenge emerged in the tension between the different worlds; the academic world can seem preoccupied with issues that don’t always feel relevant from a decision-maker perspective (and the opposite is likely also true). In the initial stages of the project, it seemed that the academic team were focused on internal processes rather than thinking about what that might mean and trying to create methodology. A larger focus on practical applications of the research, in addition to involvement early in the research activity, would incentivise decision-maker engagement.

It would also be valuable to engage decision-makers from many different levels, such as non-governmental organizations, local health authorities, and general practitioners. These organizations are often given mandates without the tools to implement or evaluate and could provide valuable advice for knowledge synthesis and producing knowledge transfer (KT) materials.

## Suggestions for Knowledge Transfer Materials: Practical Tools and Products for Decision-Makers

The ideas, strategies, and tools put together by the research team, empirically or conceptually, would be a great resource for organizations to address gaps in their attempts to achieve integrated care. KT materials also enable bottom-up improvement, which, in the context of integrated care, would avoid the high degree of complexity and necessity for strong political leadership associated with a top-down approach.

A self-assessment toolkit would allow organizations to use the research findings to self-audit in order to select appropriate improvement and transformation activities. The toolkit should include a limited, clear set of criteria assessed through both qualitative review and discussion and formal quantitative measurements, with patient- and population-oriented measurements as well as collaborative assessment measurements, such as how to recognize desired leadership at various levels (e.g. clinical or managerial). If the tool is seen as pragmatic, useful, and built from the real world, it will be readily used due to the current lack of tools available to organizations.

Useful promulgation materials for decision-makers might include short, snappy case studies that can be targeted to different audiences (e.g. operational or managerial workforces, governance, or patients and carers). Key messages and ‘mantras’ enable promulgation, as do a set of FAQs that can be presented to people who want to delve deeper into the nuances and subtleties of the material without going into the research itself. Finally, infographics using simple health literacy principles can be easily and effectively disseminated throughout networks. Such materials are useful tools to address the barriers to engagement.

It is also important to consider the factor of human skill that enables KT efforts and supplements KT materials. Any transformative effort is fighting against the constant competing paradigm of the status quo; external support, such as facilitation and/or training programs, can ensure commitment from leadership and maintain focus, and can be withdrawn when it is no longer needed.

## Looking Ahead

Research on integrated care and chronic disease management is becoming increasingly relevant for population health and systems design around the world. As ambassadors of context, decision-makers can provide a unique, real-world perspective that contextualizes the research in specific jurisdictions and policy contexts. Decision-maker engagement relies heavily on practical applications of the research findings, and efforts should be made to engage decision-makers particularly in the design and knowledge synthesis phases of the research project to optimize added value.

## Author Informations

Karen Evison is the National Programme Manager for Cardiovascular, Diabetes and Long-Term Conditions at the Ministry of Health in New Zealand. She works in the integrated service design group in the Service Commissioning Branch of the Ministry, with a focus on systems design for service improvement and reducing variation in care from individual constituent District Health Boards (DHBs).

Denis Roy is the Vice President, Science and Clinical Governance of the Institut National d’Excellence en Santé et en Services Sociaux (INESSS), an independent public agency advisory to the Minister of Health and Social Services in Quebec. The agency provides impartial, objective guidance and orientation to the Minister and professional practitioners on important policy and practice issues, with the aim of translating data, evidence, and research findings into systems change.

Fredrika Scarth is Director, Health Quality Ontario Liaison and Program Development, in the Quality and Funding Division of the Ontario Ministry of Health and Long Term Care. Her work focuses on payment reform and service delivery redesign to improve patient outcomes and experience and reduce system costs.

